# Double-scope endoscopic submucosal dissection of superficial laryngeal cancer to preserve the superior laryngeal nerve

**DOI:** 10.1055/a-2085-0774

**Published:** 2023-05-26

**Authors:** Yoshinori Horikawa, Koichi Hamada, Yoshiki Shiwa, Yusuke Mada, Kae Techigawara, Masafumi Ishikawa, Akiyoshi Ishiyama

**Affiliations:** 1 Department of Gastroenterology, Southern-Tohoku General Hospital, Koriyama, Japan; 2 Department of Minimally Invasive Surgical and Medical Oncology, Fukushima Medical University, Fukushima, Japan; 3 Department of Otolaryngology, Southern-Tohoku General Hospital, Koriyama, Japan; 4 Department of Gastroenterology, Cancer Institute Hospital, Japanese Foundation for Cancer Research, Tokyo, Japan

Endoscopic submucosal dissection (ESD) is a minimally invasive surgery for laryngeal cancers 1 , during which nerve sparing is necessary to preserve function 2 . The internal branch of the superior laryngeal nerve (ibSLN) enters the parietal recess mucosa and is distributed in the submucosa of the larynx and hypopharynx 3 . The ibSLN controls sensory perception. Damage to this may result in loss of the laryngeal cough reflex, resulting in aspiration pneumonia. A previous study reported the efficacy of double-scope ESD (ds-ESD) for laryngeal cancers 4 . We believe that ds-ESD is effective in nerve preservation. Here, we report a case of successful nerve-sparing ESD using the double-scope method.

A 72-year-old man presented with a 25 mm type 0-IIa lesion of the right piriform sinus ( Fig. 1 a ), which was surgically treated under general anesthesia ( Video 1 ). During treatment, an otolaryngologist elevated the larynx using a curved laryngoscope (Nagashima Medical Instruments Co., Ltd., Tokyo, Japan) to ensure an adequate working space. Chromoendoscopy with 0.75 % Lugol’s solution clearly revealed the lesion ( Fig. 1 b ), and marking dots were placed around the lesion using a DualKnife J (KD-655Q; Olympus, Tokyo, Japan). Cutting and dissection were performed using a transoral endoscope (GIF-H290T; Olympus). A saline solution was injected into the subepithelium. After the mucosa was cut and the subepithelial layer was dissected to a length of ≥ 5 mm for grasping ( Fig. 2 ), appropriate traction was performed using a thin transnasal endoscope (GIF-1200N; Olympus) and grasping forceps (FG-4L-1; Olympus). The traction direction was changed by controlling the angle of the thin endoscope.

**Fig. 1:**
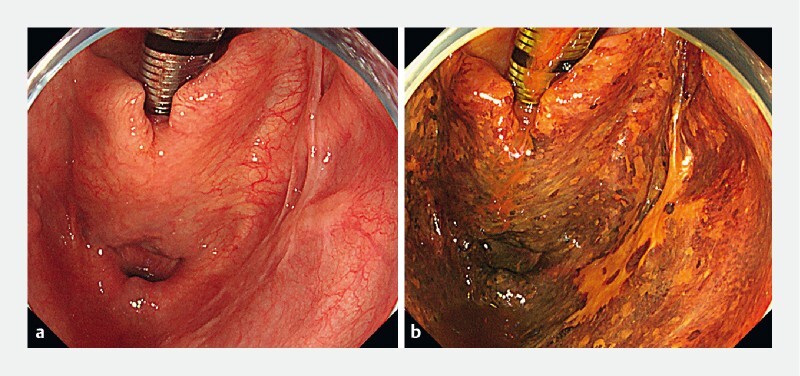
Endoscopic images. **a** White-light image of the lesion. A 25 mm type 0-IIa lesion was located at the right piriform sinus. **b** 0.75 % Lugol chromoendoscopy. The lesion could be seen more clearly than with white-light imaging.

**Video 1** Double-scope endoscopic submucosal dissection for superficial laryngeal cancer to preserve the superior laryngeal nerve.


**Fig. 2:**
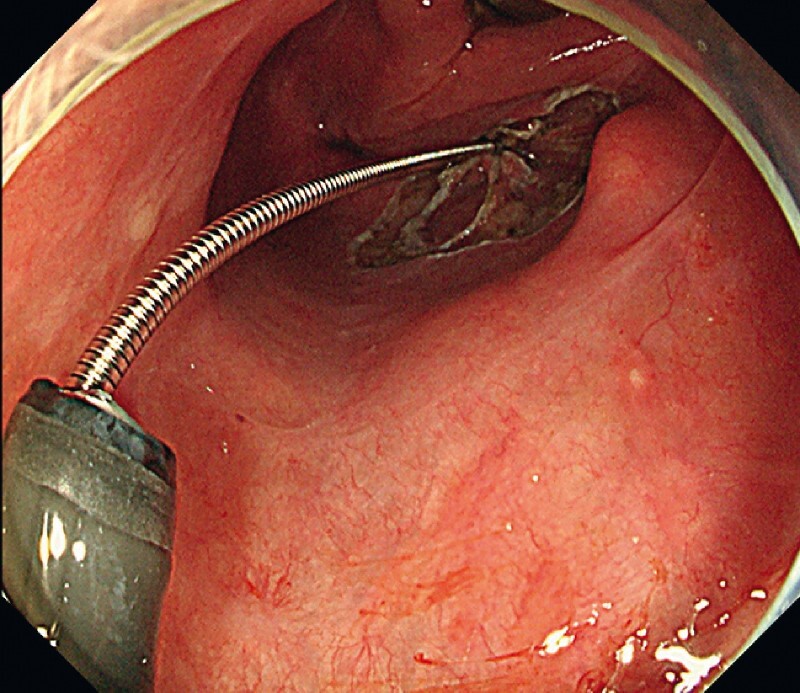
Proper traction with a transnasal thin endoscope and a grasping forceps.

Proper traction with ds-ESD allowed visualization of the ibSLN and enabled nerve-sparing ESD ( Fig. 3 ). No dysphagia or aspiration pneumonia occurred after ESD.

**Fig. 3:**
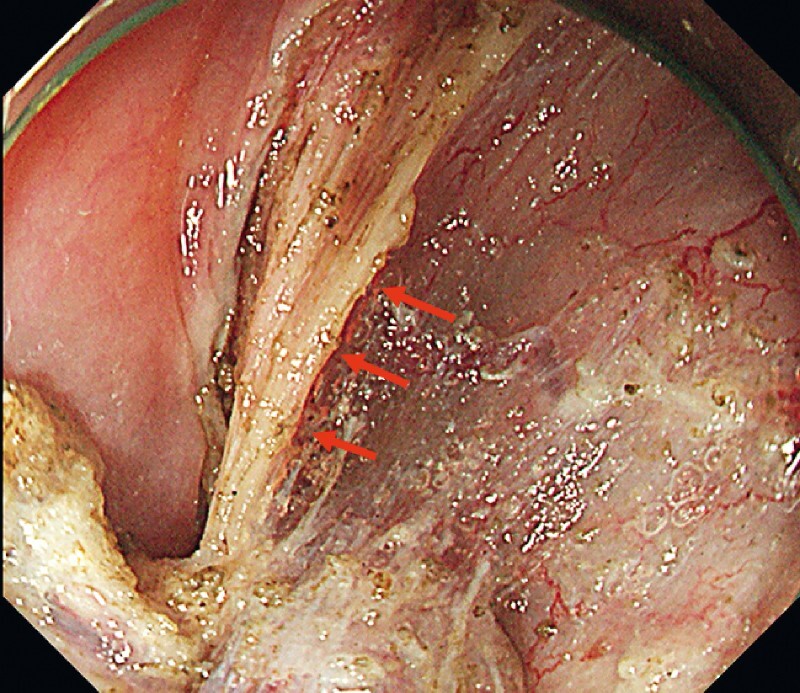
A good view of the internal branch of the superior laryngeal nerve (red arrows) was achieved with the double-scope method.

Endoscopy_UCTN_Code_CCL_1AB_2AB

## Notes

Endoscopy E-Videos

https://eref.thieme.de/e-videos
*E-Videos* is an open access online section of the journal *Endoscopy* , reporting on interesting cases and new techniques in gastroenterological endoscopy. All papers include a high-quality video and are published with a Creative Commons CC-BY license. Endoscopy E-Videos qualify for HINARI discounts and waivers and eligibility is automatically checked during the submission process. We grant 100% waivers to articles whose corresponding authors are based in Group A countries and 50% waivers to those who are based in Group B countries as classified by Research4Life (see: https://www.research4life.org/access/eligibility/ ). This section has its own submission website at https://mc.manuscriptcentral.com/e-videos
